# Effects of Drought Stress at the Booting Stage on Leaf Physiological Characteristics and Yield of Rice

**DOI:** 10.3390/plants13243464

**Published:** 2024-12-11

**Authors:** Xiaolong Yang, Xiuxiu Wang, Yang Li, Lantian Yang, Long Hu, Yuling Han, Benfu Wang

**Affiliations:** 1 Hubei Key Laboratory of Food Crop Germplasm and Genetic Improvement, Institute of Food Crops, Hubei Academy of Agricultural Sciences, Wuhan 430064, China; liylcy@163.com (Y.L.); lantian930524@163.com (L.Y.); wbfben@163.com (B.W.); 2 Institute of Agricultural Economics and Technology, Hubei Academy of Agricultural Sciences, Wuhan 430072, China; wxx17320561289@163.com; 3 Department of Biological Engineering, Hubei Vocational College of Bio-Technology, Wuhan 430070, China; hulong822@163.com; 4 Tropical Crops Genetic Resources Institute, Chinese Academy of Tropical Agricultural Sciences, Haikou 571101, China; hyl_0211@126.com

**Keywords:** rice yield, drought stress, chlorophyll, photosynthesis, antioxidant enzyme

## Abstract

Drought stress is a major environmental constraint that limits rice (*Oryza sativa* L.) production worldwide. In this study, we investigated the effects of drought stress at the booting stage on rice leaf physiological characteristics and yield. The results showed that drought stress would lead to a significant decrease in chlorophyll content and photosynthesis in rice leaves, which would affect rice yield. Three different rice varieties were used in this study, namely Hanyou73 (HY73), Huanghuazhan (HHZ), and IRAT109. Under drought stress, the chlorophyll content of all cultivars decreased significantly: 11.1% and 32.2% decreases in chlorophyll a and chlorophyll b in HHZ cultivars, 14.1% and 28.5% decreases in IRAT109 cultivars, and 22.9% and 18.6% decreases in HY73 cultivars, respectively. In addition, drought stress also led to a significant decrease in leaf water potential, a significant increase in antioxidant enzyme activity, and an increase in malondialdehyde (MDA) content, suggesting that rice activated a defense mechanism to cope with drought-induced oxidative stress. This study also found that drought stress significantly reduced the net photosynthetic rate and stomatal conductance of rice, which, in turn, affected the yield of rice. Under drought stress, the yield of the HHZ cultivars decreased most significantly, reaching 30.2%, while the yields of IRAT109 and HY73 cultivars decreased by 13.0% and 18.2%, respectively. The analysis of yield composition showed that the number of grains per panicle, seed-setting rate, and 1000-grain weight were the key factors affecting yield formation. A correlation analysis showed that there was a significant positive correlation between yield and net photosynthetic rate, stomatal conductance, chla/chlb ratio, Rubisco activity, and Fv/Fm, but there was a negative correlation with MDA and non-photochemical quenching (NPQ). In summary, the effects of drought stress on rice yield are multifaceted, involving changes in multiple agronomic traits. The results highlight the importance of selecting and nurturing rice varieties with a high drought tolerance, which should have efficient antioxidant systems and high photosynthetic efficiency. Future research should focus on the genetic mechanisms of these physiological responses in order to develop molecular markers to assist in the breeding of drought-tolerant rice varieties.

## 1. Introduction

Traditional flooding irrigation cultivation is one of the important ways of growing rice in China, whose water consumption for rice (*Oryza sativa* L.) irrigation accounts for 65% of the annual agricultural water consumption [1,2]. In recent decades, droughts have occurred frequently, intensified under global climate change, and, especially seasonal and regional droughts, severely restricted the stability of rice production in the middle and lower reaches of the Yangtze River and southern rice-growing areas in China [3,4]. Similarly, soil salinity levels are gradually increasing with the increase in drought frequency and severity, and by 2050, 50% of the world’s cultivated land will be affected by soil salinity, so increased soil salinity in arid climates further exacerbates the impact on crop production [5]. It is expected that, by the end of the century, China’s rice production will decrease by an additional 8% due to dry climate conditions caused by climate warming. This anticipated decline in rice yield is attributed to the increasingly dry climatic conditions that are expected to prevail as a consequence of global warming [6]. According to the Global Climate Change Report, the global average surface temperature has increased by 0.85 °C in the past 130 years (1880~2012), and it is expected that the global average surface temperature will continue to increase by 0.3 °C~0.7 °C from 2016 to 2035 [7]. Under the influence of high temperature, the restriction of drought stress on rice production becomes more and more obvious. Therefore, to ensure national food security, it is urgent to study the cultivation and regulation technology of rice under drought conditions to stabilize the total rice production in China [8].

In general, rice exhibits sensitivity towards water deficiency, leading to a decreased grain yield in response to drought stress. Nevertheless, variations exist in drought tolerance among rice varieties, alongside differences in the stage of growth, duration, and severity of drought stress. Consequently, these factors give rise to diverse physiological responses and agronomic characteristics among various rice varieties [3,9,10]. Drought stress may occur in the whole growth period of rice, or it may be limited to a certain growth stage, and the degree and duration of drought stress in different growth stages have different effects on rice growth and development [11,12]. The booting stage is rice’s most sensitive period to water, and drought stress causes insufficient photosynthetic capacity and reduced dry matter accumulation in the shoots in the later stage of rice production, representing important explanatory factors for the decline in yield [13,14]. Drought stress in the early stages of reproductive growth could lead to premature leaf senescence, resulting in insufficient photosynthetic productivity in the later stages and shortening the time of grain filling [15,16]. However, previous studies suggested that drought stress may hinder the occurrence of branch and spikelet development, resulting in an insufficient grain number per panicle and a shortened panicle length, representing the main reason for yield reduction [17,18]. Therefore, the mechanism of the effect of drought stress on rice yield at the booting stage needs further exploration [19].

As an important organ of rice, leaves provide various nutrient components to the grains through carbon capture and reactivation processes. If the functional leaf lifespan of rice after flowering could be extended by 1 day, the rice yield could be increased by 2% [20,21]. Under persistent drought stress conditions, the curling of the leaves gradually extends from the edge towards the tip, ultimately affecting the entire leaf. This process accelerates leaf senescence and subsequent death [10]. The damage caused by drought stress to the photosynthetic system is enacted through a complex process. Photo-carbon imbalance leads to a disruption in the equilibrium of reactive oxygen species (ROS) supply and demand, which reduces the energy distribution of the photosynthetic reaction center. This results in a reduced ability of CO_2_ to transfer from the atmosphere to carboxylation sites in the chloroplast stroma [22,23,24,25].

Previous research has primarily concentrated on the reaction mechanisms of drought stress during critical growth stages, yet there exists a notable scarcity of investigations into the premature senescence of leaves caused by drought stress. Consequently, this study aims to address this knowledge gap by utilizing rice varieties exhibiting diverse drought resistance capabilities as experimental subjects. The drought stress treatment was administered at the booting stage, and this study analyzed the alterations in agronomic and physiological traits among different varieties prior to and following the application of drought stress. The ultimate objective was to offer theoretical and practical insights for water-efficient rice cultivation in arid regions.

## 2. Results

### 2.1. Chlorophyll Content and Soil–Plant Analysis Development (SPAD) Value Induced by Drought Stress Treatment

**Figure 1:**
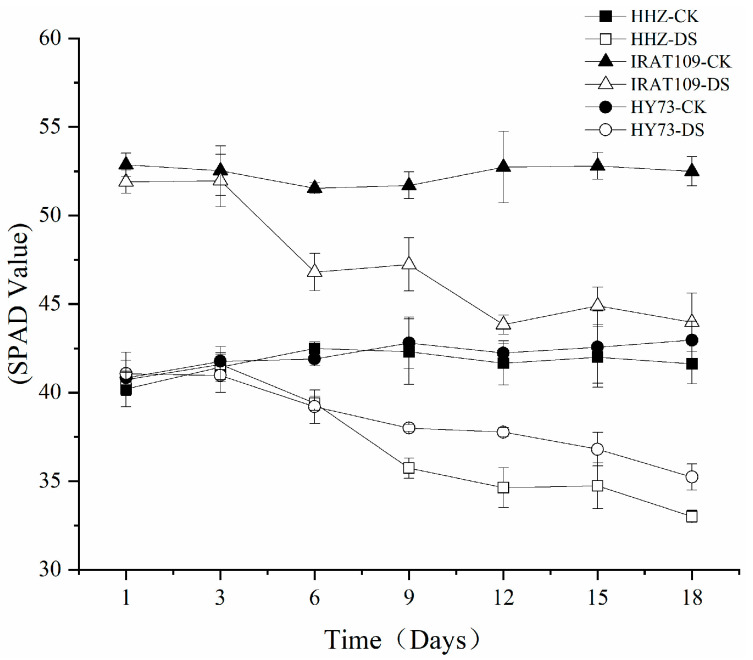
Dynamic change in leaf SPAD values after drought stress treatment (day 1 to day 18). The bars represent the standard error (SE), n = 3.

The chlorophyll content of all cultivars significantly decreased under drought stress after the booting stage (Table 1). Compared to the control check (CK) treatment, the chlorophyll a (Chl a) and chlorophyll b (Chl b) contents in HHZ cultivar plants were consistently lower by an average of 11.1% and 32.2%, respectively, while IRAT109 showed a decrease of 14.1% and 28.5%, and HY73 exhibited a decrease of 22.9% and 18.6%, respectively, under drought stress conditions. However, the carotenoid content remained unaffected by drought stress across all cultivars. Notably, the reduction in Chl a content was less pronounced than that of Chl b in all cultivars under drought stress conditions, resulting in an increase of 31.4% for HHZ cultivar plants, an increase of 20.1% for IRAT109, and an increase of 26.5% for HY73 in their respective Chl a/Chl b ratios. Rice’s ability to enhance the light-harvesting complex as an adaptive response to drought stress is evident from these findings. Similar results were observed when measuring the SPAD value of the leaves (Figure 1). The results demonstrated that the SPAD value decreased over time for all cultivars tested. On day 18 following exposure to drought stress treatment, there was no significant difference between HHZ (−18.9%), IRAT109 (−15.3%), and HY73 (−14%) with regard to the degree at which their SPAD values decreased. These findings further support the notion that drought stress leads to a reduced chlorophyll content in plants post treatment.

### 2.2. Leaf Water Potential (LWP) Induced by Drought Stress Treatment

**Figure 2:**
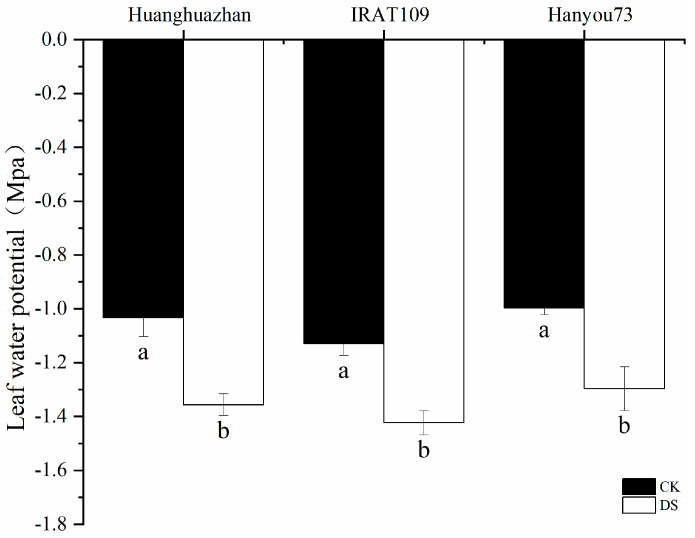
Effects of drought stress on leaf water potential (LWP) on day 9 after drought stress treatment. Different letters indicate significant differences between the treatments using Tukey’s test (*p* < 0.05). Data represent means of n = 3 measurements ± standard deviation.

Drought stress at the booting stage led to significant leaf water loss. On day 9 of the drought stress treatment, the LWP decreased significantly in HHZ by 31.3% (from −1.03 Mpa in non-drought-treated plants to −1.36 Mpa in drought-treated plants). Correspondingly, the LWP decreased significantly in IRAT109 and HY73 by 29.6% (from −1.13 Mpa in non-drought-treated plants to −1.42 Mpa in drought-treated plants) and 30.1% (from −1.00 Mpa in non-drought-treated plants to −1.30 Mpa in drought-treated plants), respectively (Figure 2). There was no significant difference in the degree of decrease in LWP between the three cultivars.

### 2.3. Antioxidant Enzyme Activities and Malondialdehyde (MDA) Content Induced by Drought Stress Treatment

An analysis of variance showed a significant main effect (*p* < 0.01) of the cultivar and drought treatment on the accumulation of antioxidant enzyme activity and MDA (Table 2). Drought stress at the booting stage led to a significant enhancement in the antioxidant enzyme system and the occurrence of membrane peroxidation. On day 9 of the drought stress treatment, superoxide dismutase (SOD), peroxidase (POD), and catalase (CAT) activities significantly increased by 30.3%, 38.3%, and 36.3% in HHZ, 15.2%, 19.4%, and 24.8% in IRAT109, and 23.9%, 34.9%, and 33.9% in HY73, respectively. The content of MDA increased by 28.2% in HHZ, 18.8% in IRAT109, and 18.7% in HY73. In IRAT109, the degree of decrease in the activity of the antioxidant enzyme system was lower than in HHZ and HY73, and similar results were found in MDA. The results indicated that IRAT109 might be better than the other two varieties in terms of drought resistance. These results show that drought stress led to increased antioxidant enzyme activities and MDA content in the plants after the treatments. It is generally expected that the activities of antioxidant enzymes such as SOD, CAT, and POD would exhibit an inverse relationship with MDA levels under stress conditions, as they are involved in mitigating oxidative damage. However, in our study, we observed a similar trend, which could be attributed to specific environmental or experimental conditions. The main reason for this was that our sampling time fell on the ninth day after drought stress, potentially before reaching the critical point of activity decline in the antioxidant enzyme active substances in the leaves [26,27].

### 2.4. Photosynthetic Enzyme Activities Induced by Drought Stress Treatment

**Figure 3:**
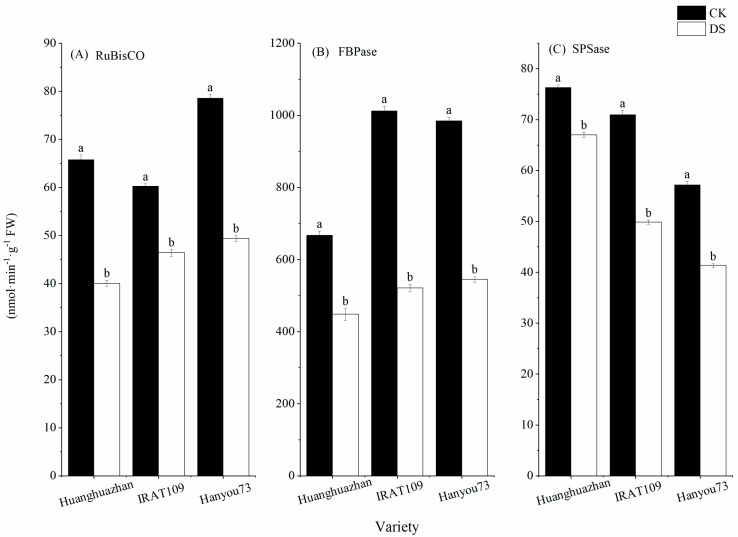
Effects of drought stress on Rubisco, FBPase, and SPSase activities on day 9 after drought stress treatment: (**A**) ribulose bisphosphate carboxylase; (**B**) fructose-1,6-diphosphatase; and (**C**) sucrose phosphate synthase. Different letters indicate significant differences between the treatments using Tukey’s test (*p* < 0.05). Data represent means of n = 3 measurements ± standard deviation.

Drought stress treatment at the booting stage led to a significant decrease in photosynthetic synthase activities. On day 9 of the drought stress treatment, the ribulose bisphosphate carboxylase (Rubisco), fructose-1,6-diphosphatase (FBPase), and sucrose phosphate synthase (SPSase) activities were decreased by 39.1%, 32.8%, and 12.2% in HHZ, 22.9%, 48.5%, and 29.7% in IRAT109, and 37.2%, 44.7%, and 27.7% in HY73, respectively (Figure 3). In HY73, the decrease in the activity of Rubisco was lower than in HHZ and IRAT109, and similar results were found for FBPase and SPSase.

### 2.5. Chlorophyll Fluorescence Parameters Induced by Drought Stress Treatment

**Figure 4:**
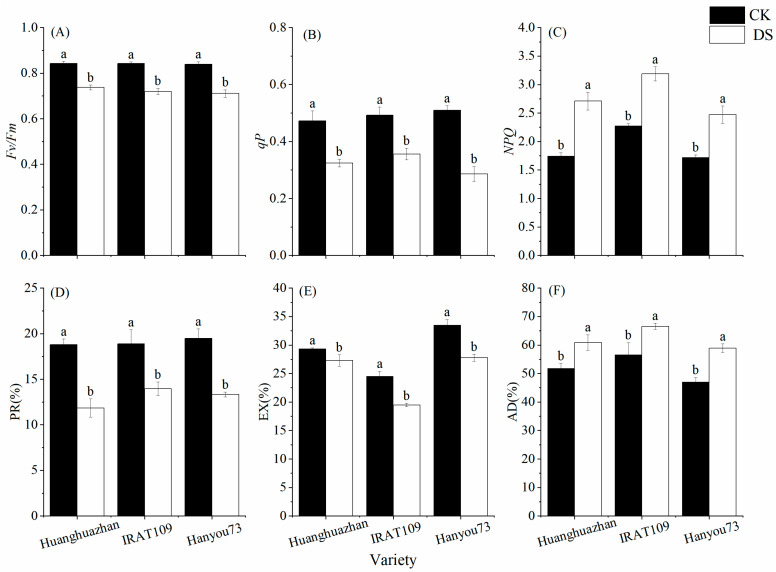
Effects of drought stress on Fv/Fm, q^p^, NPQ, and distribution of light energy PR, EX, and AD on day 9 after drought stress treatment. Different letters indicate significant differences between the treatments using Tukey’s test (*p* < 0.05). (**A**) Maximum photochemical values of PSII in the dark; (**B**) photochemical quenching; (**C**) non-photochemical quenching; (**D**) photochemical reaction; (**E**) non-photochemical reaction dissipation; and (**F**) antenna heat dissipation. Data represent the means of n = 3 measurements ± standard deviation.

The chlorophyll fluorescence parameters of rice plants were influenced by drought stress at the booting stage. The maximum photochemical values of PSII in the dark (Fv/Fm) and photochemical quenching (q^p^) of rice plants on day 9 of the drought stress treatment were significantly decreased, while the NPQ was significantly increased, and a similar trend was found in all cultivars (Figure 4). Fv/Fm and *q^p^* were decreased by 12.6% and 31.4% in HHZ, 14.5% and 27.7% in IRAT109, and 15.3% and 43.9% in HY73, respectively. However, NPQ was increased by 55.2% in HHZ, 40.0% in IRAT109, and 43.5% in HY73, respectively (Figure 4A–C). Correspondingly, we analyzed the proportion of light energy distribution in the photosynthetic system, and a corresponding pattern was found. The results showed that photochemical reaction (PR) and non-photochemical reaction dissipation (EX) were decreased by 36.9% and 7.0% in HHZ, 26.1% and 20.5% in IRAT109, and 31.6% and 17.1% in HY73, respectively. However, the antenna heat dissipation (AD) was increased by 17.4% in HHZ, 17.6% in IRAT109, and 25.3% in HY73, respectively (Figure 4D–F). The results indicate that drought stress can reduce the photochemical activity, energy conversion efficiency, and light amount of leaves, eventually inhibiting leaf photosynthesis.

### 2.6. Photosynthetic Rate Induced by Drought Stress Treatment

**Figure 5:**
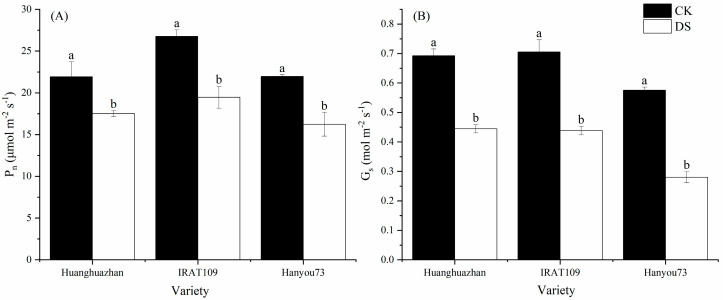
Effects of drought stress on net photosynthetic rate and stomatal conductance (G_s_) on day 9 after drought stress treatment: (**A**) net photosynthetic rate and (**B**) stomatal conductance. Different letters indicate significant differences between the treatments using Tukey’s test (*p* < 0.05). Data represent the means of n = 3 measurements ± standard deviation.

**Figure 6:**
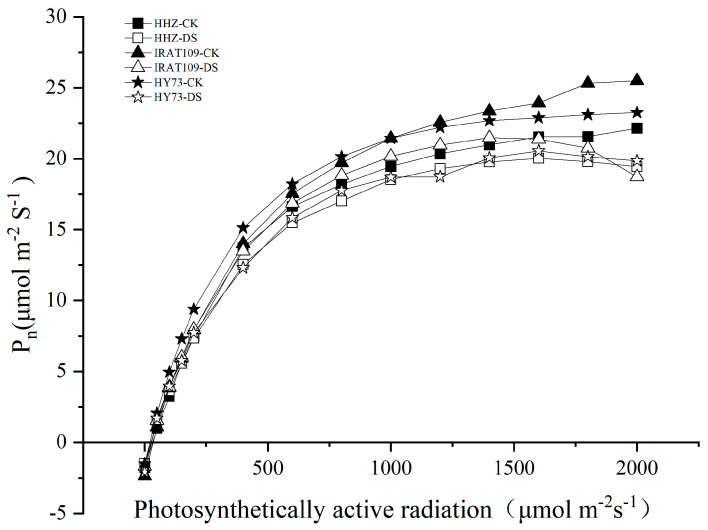
Effects of drought stress on photosynthetic light response curve on day 9 after drought stress treatment.

The photosynthetic rate (P_n_) of rice plants was significantly decreased by 20.1% (from 21.9 μmol·m^−2^·s^−1^ in non-drought-treated plants to 17.5 μmol·m^−2^·s^−1^ in drought-treated plants) in HHZ, 27.3% (from 26.7 μmol·m^−2^·s^−1^ in non-drought-treated plants to 19.5 μmol·m^−2^·s^−1^ in drought-treated plants) in IRAT109, and 26.1% (from 22.0 μmol·m^−2^·s^−1^ in non-drought-treated plants to 16.2 μmol·m^−2^·s^−1^ in drought-treated plants) in HY73, respectively (Figure 5A). Similar to the variation trend in the photosynthetic rate, the stomatal conductance (G_s_) of rice plants also decreased significantly under drought treatment, specifically by 35.8% in HHZ, 37.8% in IRAT109, and 51.3% in HY73, respectively (Figure 5B). The results of the light response curve show that the net photosynthetic rate of plants increased significantly with the increase in light intensity. When the photosynthetically active radiation (PAR) ≤ 400 μmol·m^−2^·s^−1^, there was no significant difference in leaf P_n_ between the three rice cultivars. With the increase in PAR intensity, the degree of increase in the P_n_ of drought-treated plants was lower than in non-drought-treated plants. When PAR reached 1300 μmol·m^−2^·s^−1^, the P_n_ of drought-treated plants tended to be stable; however, when PAR exceeded 1800 μmol·m^−2^·s^−1^, the P_n_ of drought-treated plants decreased significantly, while the photosynthetic rate of untreated plants increased slightly (Figure 6). This suggests that photosynthesis in rice under drought stress may be affected by photoinhibition.

### 2.7. Yield and Yield Components Induced by Drought Stress Treatment

Compared to the CK sample, the rice grain yield exhibited a significant decline (*p* < 0.05) of 30.2% in HHZ, 13.0% in IRAT109, and 18.2% in HY73, respectively (Table 3). The magnitude of yield reduction among the three rice cultivars followed the order of HHZ > HY73 > IRAT109. Yield component analysis revealed that the number of spikelets per panicle, seeding rate, and 1000-grain weight significantly influenced yield formation under the drought stress treatment. However, drought stress treatment did not have a significant effect on effective panicle development in these cultivars. In HHZ, the primary reason for a decreased yield due to drought treatment was a substantial decrease in spikelet number per panicle, seeding rate, and 1000-grain weight. Compared to the control group, there was a reduction of 16.7% in the spikelet number per panicle, a decrease of 15.8% in the seeding rate, and a decline of 5.7% in the thousand-grain weight. For IRAT109, the main causes for a reduced yield after drought treatment were significant decreases in spikelet number per panicle and thousand-grain weight compared to the control group, with reductions of approximately 9.7% and 4.9%, respectively. In a similar vein, regarding the HY73 cultivar and its performance under drought conditions, the primary reasons for its diminished yield could be attributed to significant declines in both the number of spikelets per panicle and the seeding rate. These reductions were observed when compared with the control group, with the spikelet number per panicle decreasing by approximately 12.1% and the seeding rate experiencing a reduction of around 10.6%. The outcomes from the analysis of variance suggested that there was a crucial interaction between the treatment that was applied and the specific cultivar that was utilized. This interaction played a pivotal role in determining the overall response of the crop to drought conditions, highlighting the importance of considering both the type of treatment and the cultivar when aiming to understand and mitigate the effects of drought on crop yield.

### 2.8. Multivariate Statistical Analysis

**Figure 7:**
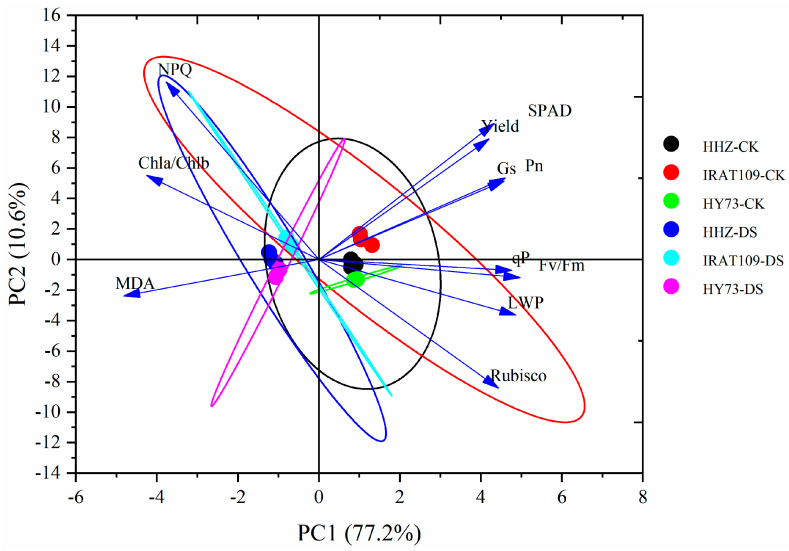
Principal component analysis of all three cultivars under drought stress at the booting stage. HHZ-CK: flooding irrigation of Huanghuazhan; IRAT109-CK: flooding irrigation of IRAT109; HY73-CK: flooding irrigation of Hanyou73; HHZ-DS: drought stress of Huanghuazhan; IRAT109-DS: drought stress of IRAT109; and HY73-DS: drought stress of Hanyou73.

**Figure 8:**
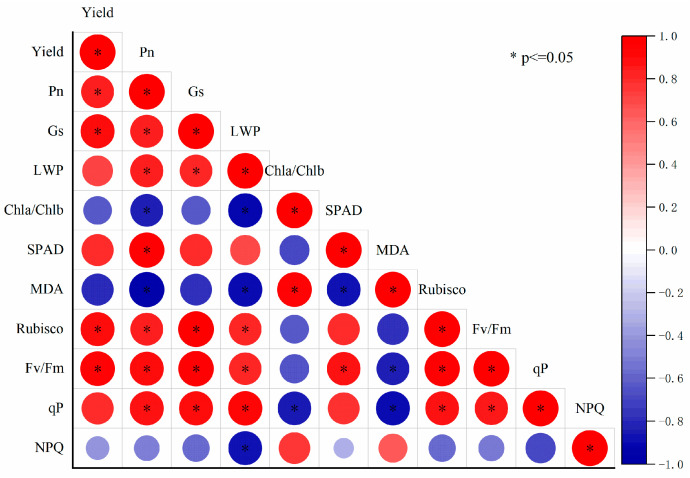
Correlation analysis among 11 physiological indexes of all three cultivars under drought stress at the booting stage. Pn: net photosynthetic rate; Gs: stomatal conductance; LWP: leaf water potential; Chla/Chlb: ratio of chlorophyll a to chlorophyll b; SPAD: soil–plant analysis development value; MDA: malondialdehyde; Rubisco: ribulose bisphosphate carboxylase; Fv/Fm: maximum photochemical value of PSII in the dark; qP: photochemical quenching; and NPQ: non-photochemical quenching. * *p* < 0.05.

The selection of principal components was based on the eigenvalues and contribution rates. The physiological and biochemical indexes of the leaves from the three rice cultivars under drought stress at the booting stage were analyzed by PCA. Two principal components with eigenvalues greater than 1 were obtained, and their contribution rates were 77.21% and 10.59%, respectively. The cumulative contribution rate was 88.7%, and most of the information on the original characteristics was retained (Table 4). Therefore, the first two principal components could be selected as the important principal components of the drought effects on leaf physiological characteristics and yield. The factors with a higher loading capacity in the first principal component were SPAD, P_n_, G_s_, qP, Fv/Fm, LWP, and Rubisco, which were mainly related to leaf water status, chlorophyll content, photosynthetic enzyme, and photosynthesis. The second principal component was mainly related to osmotic solutes and light energy distribution. To further understand the relationship between these parameters, Pearson’s correlation analysis was used to analyze the data (Figure 7). The correlation analysis revealed a significant positive relationship between grain yield and P_n_, G_s_, Rubisco, and Fv/Fm. Conversely, negative correlations were observed between grain yield and MDA as well as Chl a/Chl b and NPQ (Figure 8). The trait of P_n_ had a positive and significant correlation with traits of G_s_, LWP, SPAD, Rubisco, Fv/Fm, and q^p^, and there was a negative and significant correlation with the trait of MDA and Chl a/Chl b. The G_s_ trait showed a positive and significant correlation with LWP, Rubisco, Fv/Fm, and q^p^, and there was a negative correlation with the trait of MDA, Chl a/Chl b, and NPQ. Also, the LWP trait had a positive and significant correlation with Rubisco, Fv/Fm, and q^p^, and there was a negative correlation with the trait of MDA, Chl a/Chl b, and NPQ. The Chl a/Chl b trait showed a positive and significant correlation with MDA, and there was a negative correlation with the trait of q^p^. For the trait of SPAD, there was a positive and significant correlation with Fv/Fm, and a negative and significant correlation with the trait of MDA. The MDA trait showed a negative and significant correlation with the trait of Fv/Fm and q^p^. The Rubisco trait showed a positive and significant correlation with the trait of Fv/Fm and q^p^. Also, the Fv/Fm trait showed a positive and significant correlation with the trait of q^p^. The results of the Pearson correlation analysis were further supported by the principal component analysis, which grouped the variables into distinct clusters based on their inter-relationships. The first principal component was heavily influenced by the SPAD and Fv/Fm values, indicating their strong association with the photosynthetic efficiency. The second principal component was characterized by MDA and NPQ, suggesting a link between oxidative stress and the electron transport rate in the photosynthetic process. These findings provided a comprehensive understanding of the interplay between various physiological traits and their impact on plant performance under stress conditions.

## 3. Discussion

Drought stress is a major factor affecting the development of rice production globally With elevating ambient drought stress, the negative effects of drought on the growth and development of rice are becoming increasingly obvious, especially in crop photosynthesis and yield [10,28,29]. Our study aimed to investigate the effects of drought stress on rice leaf physiology and their correlation with yield traits. The results obtained provide valuable insights into the mechanisms underlying drought tolerance in rice.

Photosynthesis is the foundation of crop yield, and improving the photosynthetic efficiency of rice under adverse conditions is an important means to stabilize food production. However, the impact of drought stress during the booting stage on the photosynthesis of rice leaves can persist into the late growth stages, which is mainly reflected by G_s_, chlorophyll content and LWP [3,30,31]. In this study, we noted that there were considerable changes in a range of physiological characteristics when plants were subjected to drought stress. More specifically, the drought stress caused by a lack of water led to a notable decrease in the conductance of stomata (Figure 5). Additionally, we also observed a reduction in the content of chlorophyll (Table 1) and a decline in the leaf water potential (Figure 2). This indicated that the decrease in G_s_ limited the diffusion of CO_2_ in the leaf, thereby reducing the rate of photosynthesis [32]. The reduction in chlorophyll content suggested that the photosynthetic apparatus was damaged. In addition, the increase in the ratio of Chla/Chlb could have potentially resulted in an imbalance in the absorption of particular wavelengths of light, further confirming the negative impact of drought on photosynthesis (Figure 8) and, in turn, compromising the plant’s ability to capture light energy [33]. Therefore, reducing the ratio of Chla/Chlb in leaves under drought conditions is an important indicator for maintaining a stable photosynthetic rate. Moreover, a lower LWP indicates that the plant in question is experiencing water stress, which could lead to a decrease in cell turgor and affect the overall physiological functions of the leaf. Early studies suggested that the carbon assimilation rate decreases due to the insufficient water requirement of leaves [34,35]. These findings collectively suggest that drought stress has a profound impact on vital processes in plant physiology, affecting both the efficiency of photosynthesis and a plant’s ability to regulate water loss through its stomata.

To further elucidate the effects of drought stress on rice physiology, we also examined the changes in key photosynthetic enzymes and antioxidant enzymes. The major sites of ROS production in the chloroplasts of photosynthetic plants are photosystem II (PSII) and photosystem I (PSI), which impede photosynthesis and induce photoinhibition [36]. Our findings revealed that on day 9 after the drought stress treatment, the activities of SOD, POD, and CAT significantly increased (Table 2), while the activities of Rubisco, FBPase, and SPSase were significantly reduced (Figure 3) compared to the control plants. These results showed that photosynthesis was inhibited under drought stress, which might have been due to the production and accumulation of a large number of ROS in the leaves. The production and accumulation of ROS in the plants resulted in the severe destruction of cell organelles and functions, causing membrane peroxidation and leading to damage in the cell membrane, the degradation of biological macromolecules, and, ultimately, cell death [37,38]. However, the decrease in stomatal conductance interferes with the intake of CO_2_, which alters enzymatic activities, causes membrane disruption, and reduces ATP synthesis and Rubisco regeneration, thus inhibiting Rubisco activity and affecting the process of photosynthesis [39,40]. This imbalance between the production of ROS and a plant’s defense mechanisms could ultimately lead to a decline in plant growth and yield under drought conditions. In addition, our analysis of chlorophyll fluorescence kinetic parameters revealed a decrease in PR and EX in all three cultivars (Figure 4). However, AD exhibited increases of 17.4%, 17.6%, and 25.3%, respectively, in HHZ, IRAT109, and HY73. Increases in non-photochemical quenching (Figure 4) help dissipate excess light energy as heat, protecting the photosynthetic apparatus under drought conditions. Previous studies have shown that drought stress leads to a decrease in the energy allocated to the photosynthetic reaction center, causing electron transport to be hindered [41]. The results of this study indicate that, when plants are subjected to drought stress, there is a notable decrease in the photochemical activity within their leaves. This means that the ability of the leaves to convert light energy into chemical energy is compromised [42]. Additionally, the efficiency of energy conversion is significantly diminished, further contributing to the overall stress response. Moreover, the capacity of the leaves to intercept and utilize light is also adversely affected [43]. These combined factors ultimately result in a substantial inhibition of the photosynthetic processes, which are crucial for the plant’s ability to produce food and grow [44]. These physiological changes are consistent with the observed reduction in grain yield and its components, as reported in our study (Figure 8).

The production of rice fundamentally relies on various yield components, and any changes or alterations in these components, particularly those induced by drought stress during the booting stage of rice development, play a crucial role in determining the ultimate grain yield [10,19]. In terms of yield traits, our study found that drought stress significantly reduced rice grain yield in all three cultivars, with HHZ experiencing the most substantial decline of 30.2%, followed by HY73 at 18.2% and IRAT109 at 13.0% (Table 3). This variance in yield reduction suggests that rice cultivars have differing levels of drought tolerance. HHZ appears to be the least resilient to drought conditions, while IRAT109 demonstrates a higher level of tolerance. Positive results with a decrease in grain yield under drought stress at the booting state were reported, which were associated with a decrease in the number of spikelets per panicle, seeding rate, and 1000-grain weight [45]. For HHZ, the primary factor contributing to yield reduction was the decrease in the number of spikelets per panicle, seeding rate, and 1000-grain weight. For IRAT109, the significant yield reduction was mainly due to a decline in the number of spikelets per panicle and 1000-grain weight. For HY73, the drought conditions led to significant decreases in both the spikelet number per panicle and the seeding rate. In the long-term process of evolution and adaptation to the geographical environment, each cultivar achieved a different level of drought resistance [4]. This study also highlighted the fact that the drought tolerance of HHZ was superior to that of IRAT109 and HY73, as it exhibited a lesser reduction in grain yield under similar stress conditions. This could be attributed to the genetic makeup of HHZ, which may possess certain adaptive traits which allow it to better maintain its yield components under water-limited environments. A multivariate statistical analysis showed that cultivars that maintained higher stomatal conductance, leaf water potential, photosynthetic rates, ribulose, and Fv/Fm under drought stress also exhibited better yield performance (Figure 7 and Figure 8). Maintaining the equilibrium and stability of physiological traits in the face of drought conditions is a crucial factor in ensuring and enhancing the productivity of grain crops [46]. Future research could explore the genetic mechanisms behind drought tolerance in rice and investigate other yield components or external factors that might influence the cultivars’ response to drought [10]. The results of our study highlight the importance of selecting and breeding rice cultivars with superior drought tolerance mechanisms, such as efficient antioxidant systems and a high photosynthetic efficiency. Future research should focus on identifying and incorporating these traits into existing rice-breeding programs to develop new varieties that can sustain productivity under scenarios with increasing drought stress. Additionally, understanding the genetic basis of these physiological responses will be crucial for developing molecular markers to assist in breeding drought-tolerant rice varieties [47,48].

## 4. Materials and Methods

### 4.1. Site Description

Pot experiments were conducted in a greenhouse at the Institute of Food Crops, Hubei Academy of Agricultural Sciences, Hubei Province, China (30°29′ N, 114°18′ E), during rice growth season of 2021 (May to October). Typical loam soil was collected from a rice paddy field located in Wuhan, China. After sieving through a 2 mm fine mesh, the soil and sand were mixed at a ratio of 4:1 and then 18 kg of sand-and-soil mixture was placed in a plastic pot (30 cm × 30 cm × 26 cm) to 1.50 g cm^–3^ of bulk density. The pH, total nitrogen, available phosphorus, potassium, and organic matter values of soil were 6.08, 0.79 g·kg^−1^, 28.2 mg·kg^−1^, 173 mg·kg^−1^, and 1.30 g·kg^−1^, respectively.

### 4.2. Experimental Design and Management

In this study, the experiment was laid out in a randomized complete block design using 15 replicates. Two water treatments were set up in the experiment—flooding irrigation during the entire growth period (CK) and drought stress at the booting stage (DS)—with three different genotype rice cultivars, Hanyou73 (HY113), Huanghuazhan (HHZ), and IRAT109, in 2021. For flooding irrigation, 1–5 cm water level was kept during the whole growth season until 1 week before harvest. For the drought stress treatment, a soil tensiometer was installed 15–20 cm below the soil surface. Data were collected at 18:00 pm every day. According to the statistical analysis of the data, water was supplied to ensure that the soil water potential was maintained at −30 kPa ± 5 kPa during the drought stress period. Irrigation after drought stress at the booting stage was consistent with the control.

Three twenty-day-old seedings of the same size were selected from the tray and manually transplanted with soil into each pot. The drought treatment was initiated on the 35th day post transplantation, and the drought stress treatment continued for a duration of 18 days. Throughout the period of drought stress treatment, the soil water potential was monitored using a soil tensiometer. Rehydration occurred when the soil water potential dropped below −35 kPa, while drainage measures were implemented when the soil water potential exceeded −25 kPa to ensure plant survival under drought conditions. The prevention and control measures of diseases, insects, and weeds were implemented in accordance with the requirements of conventional cultivation. The plants were manually harvested on 1–2 October.

### 4.3. Chlorophyll Content and Leaf Water Potential (LWP)

The chlorophyll content was extracted from the uppermost fully expanded leaves with 80% acetone, and chla, chlb, and Car were determined through a UV-1900 spectrophotometer (Shimadzu, Japan), as described in [49]. Ten of the uppermost mature leaves from each replicate were analyzed using the SPAD-502 chlorophyll meter (Konica-Minolta, Tokyo, Japan). The leaf water potential (LWP) was measured with the Psypro Dewpoint Potentia Meter (Psypro, Wescor Inc., Paris France). We selected non-destructively fully expanded functional leaves (those for the gas exchange measurements), clipped onto the probe, and used sealant seal; then, after 45 min, we determined the LWP at midday, between 12:00 and 14:00 [50].

### 4.4. Measurement of Antioxidant Enzyme Activities and MDA Accumulation

The leaves whose photosynthesis activity had been measured were taken, immediately placed in liquid N_2_, and stored in a −80 °C refrigerator. A total of 0.5 g of fresh treated and non-treated leaves was ground in liquid N_2_ using a pre-cooled mortar and homogenized in 2 mL of chilled potassium phosphate buffer in 50 mM samples (pH 7.8) with 0.5% (*v*/*v*) Triton X-100 and 1% (*w*/*v*) polyvinylpyrrolidone for extracting the crude enzymes in the powder. The homogenate was centrifuged at 18,000× *g* for 20 min, and the supernatant was taken for enzyme activity measurement [51]. Superoxide dismutase (SOD) activity was measured by inhibiting the photoreduction of nitro-blue tetrazolium (NBT) and absorbance was taken at 560 nm. Peroxidase (POD) activity was assayed through the guaiacol method, leaving POD to oxidize guaiacol to form a dark brown substance and measuring the contents of this product using a spectrophotometer at 470 nm. As catalase (CAT) can decompose hydrogen peroxide, the absorbance of the reaction solution at 240 nm decreases with the reaction time. As such, its activity was measured by the rate of change in absorbance at 240 nm. The malondialdehyde (MDA) content was measured using thiobarbituric acid [52].

### 4.5. Assays of Rubisco, FBPase, and SPSase Enzyme Activities

Rubisco activity was calculated spectrophotometrically based on the amount of nicotinamide adenine dinucleotide (NADH_2_) consumed by the reduction of 3-phosphoglyceric acid (3-PGA) whose formation had been catalyzed by Rubisco [53]. The initial activity of Rubisco was measured at 30 °C, and 0.1 mL of enzyme extract was added to the activity assay solution, which totaled 2.8 mL and contained 0.2 mmol/L NADH, 5 mmol/L ATP, 5 mmol/L Cr-P, 10 mmol/L NaHCO_3_, 50 mmol/L Hepes-KOH buffer (pH8.0), 1 mmol/L EDTA, 20 mmol/L MgCl_2_, 5 mmol/L DTT, 10 U/mL creatine phosphokinase, 10 U/mL glycerate phosphokinase, and 10 U/mL glyceraldehyde phosphokinase). Then, we added 0.1 mL RUBPcase (0.6 mmol/L) to the cuvette, started reading, and recorded the 340 nm OD value every 15 s until 1 min had passed. The enzyme activity was calculated as the number of μmol of NADH reduced per milliliter of crude enzyme solution per minute.

A total of 40 μL of the enzyme extract was added to 752 μL of the reaction solution pre-incubated at 30 °C; the reaction solution contained 30 mmol/L Hepes-KOH buffer (pH 8.2), 5 mmol/L MgCl_2_, 5 mmol/L DTT, 0.5 mmol/L NADP, 2 U/mL G6PD, and 2 U/mL PGI. Then, 8 μL 300 mmol/L FBP was added to initiate the reaction. The activity of fructose-1,6-diphosphatase (FBPase) was calculated by measuring the change in absorbance value at 340 nm within a 1 min time window, using a UV spectrophotometer [54].

Sucrose phosphate synthase (SPSase) catalyzes fructose 6 phosphate to form sucrose phosphate, and the reaction between sucrose phosphate and resorcinol can show a color change, with a characteristic absorption peak at 480 nm and enzyme activity proportional to the color depth [55]. To calculate this, we took about 0.5 g of pre-cooled rice leaves (removing the main leaf veins), added 4 mL of buffer (pH 7.0), quickly ground this into a paste in a pre-cooled mortar in an ice bath, and placed the product into a centrifuge tube. The supernatant was obtained by centrifugation at 10,000× *g* for 30 min in a low-temperature (4 °C) refrigerated centrifuge. Fructose-6 phosphate, UDPG, 0.1 mol/L Tris, 10 mmol/L MgCl_2_, and the supernatant were added to the reaction solution. We added 100 µL enzyme solution to 0.15 mL of reaction medium containing 50 mmol/L Tris-HCl, pH7.0, 10 mmol/L MgCl_2_, 10 mmol/L fructose-6 phosphate, and 3 mmol/L UDGP, and we place the mixture in a water bath at 30 °C to react for 10 min. Then, we added 2 mol/L NaOH 0.05 mL after cooling, put it in a boiling water bath for 10 min, cooled it with running water, added 1.5 mL of concentrated hydrochloric acid and 0.5 mL of 1% resorcinol, shook the product well, and finally put it in a water bath at a constant temperature of 80 °C for 10 min, conducting our colorimetric measurements at a 480 nm wavelength after cooling.

### 4.6. Leaf Gas Exchange Parameters and Chlorophyll Fluorescence

The chlorophyll fluorescence parameters include the minimum fluorescence level (F0), the maximum fluorescence level (*Fm*), and steady-state fluorescence (Fs), which were measured using a portable photosynthesis system along with a 6400-40 leaf chamber fluorometer (LI-6400 XT; Li-Cor, Lincoln, NE, USA) after dark adaption for 20 min. The light-adapted initial fluorescence (F0′) and rapid light response curves of the maximum fluorescence under light (Fm′) were measured from 9:00 am to 11:00 am on a sunny day [56]. The maximum photochemical values of PSII in the dark (Fv/Fm), photochemical quenching (q^p^), and non-photochemical quenching (NPQ) were calculated using the following formula:

Fv/Fm = (Fm − F0)/Fm; q^p^ = (Fm′ − Fs)/(Fm′ − F0′); NPQ = (Fm − Fm′)/Fm

The distribution of the light energy absorbed by plants is mainly determined by the photochemical reaction (PR), antenna heat dissipation A(D), and non-photochemical reaction dissipation (EX) for the reaction center. The calculation formula is as follows:

PR = (Fm′ − F0′)/Fm′ × q^p^;

AD = 1 − (Fm′ − F0′)/Fm′;

EX = (Fm′ − F0′)/Fm′ × (1 − q^p^)

The leaf gas exchange parameters include the net photosynthetic rate (P_n_) and the stomatal conductance (G_s_), which were measured with the LI-6400XT portable photosynthesis measurement system (Li-Cor, Lincoln, NE, USA) from 9:00 am to 11:00 am on a sunny day [57]. The photosynthesis measurement equipment was kept open, the airflow rate in the sample room was 500 μmol·s^−1^, and the light intensity of the artificial light source was set to 1200 μmol·m^−2^·s^−1^. The light response curve of flag leaves was measured using LI-6400XT with a carbon dioxide cylinder of 400 μmol·m^−2^·s^−1^ after the drought had ended. The photosynthetically active radiation (PAR) was altered between 2000, 1800, 1600, 1400, 1200, 1000, 800, 600, 400, 200, 150, 50, and 0 μmol·m^−2^·s^−1^.

### 4.7. Grain Yield and Its Components

At the maturity stage, five representative pots of unsampled plants were harvested for grain yield determination and the analysis of yield components. Each pot of rice was threshed by hand and left to dry under natural conditions. Then, impurities and shriveled particles were removed using a wind separator and weighed. Then, the moisture content of rice was measured by a grain moisture meter (DMC-700, Seedburo, Chicago, IL, USA), and the yield was calculated according to the standard moisture content of 13.5% for indica and 14.5% for japonica. The water separation method was used to separate the full grains from the empty ones and weigh them after drying. We weighed three 30 g samples from the group of saturated grains and three 2 g samples comprising only empty grains. We manually counted the number of saturated grains and empty grains in each small sample and then placed them in an oven at 80 °C to dry to a constant weight. We then used a balance with an accuracy of 0.001 g to determine the dry weight. Finally, the calculation of the yield components of effective panicle, spikelet number per panicle, seed-setting rate, and thousand-grain weight was completed.

### 4.8. Data Analysis

The data analysis and correlation coefficients were estimated with SPSS15.0 (IBM, Armonk, NY, USA) statistical software, whilst the differences between treatments were determined using the least significant difference (LSD) test at a 0.05 level of significance.

## 5. Conclusions

In conclusion, the impact of drought on rice yield is multifaceted, affecting various agronomic traits which collectively determine the productivity of the crop. In this study, the physiological functions of rice leaves, including leaf water potential and chlorophyll content, were significantly impaired by drought stress during the booting stage. This led to a decrease in the photosynthetic rate caused by the reduced activity of photosynthetic enzymes, which ultimately resulted in a decline in yield. The drought tolerance of various cultivars varied during the booting stage. HHZ appeared to be the least resilient to drought conditions, while IRAT109 demonstrated a higher level of tolerance. Moreover, the significant yield reductions observed in all three cultivars after drought stress emphasize the importance of selecting for traits, such as spikelet number per panicle, that are crucial for maintaining yield under adverse conditions. The correlation of physiological traits such as stomatal conductance, leaf water potential, and photosynthetic rates with yield performance under drought stress highlights the importance of these parameters in breeding drought-tolerant rice varieties. Future research should focus on elucidating the genetic mechanisms that underpin these physiological responses to drought. This knowledge will be instrumental in developing molecular markers that can facilitate the selection of drought-tolerant rice lines with improved yield potential.

## Figures and Tables

**Table 1:** Effects of drought stress on chlorophyll content and carotenoid on day 9 after drought stress treatment.

Cultivar	Treatment	Chl a (mg·g^−1^)	Chl b (mg·g^−1^)	Caro (mg·g^−1^)	Chl a/Chl b
HHZ	CK	1.42 ± 0.03 a	0.48 ± 0.04 a	0.31 ± 0.01 a	2.97 ± 0.19 b
	DS	1.26 ± 0.01 b	0.32 ± 0.04 b	0.30 ± 0.01 a	3.90 ± 0.37 a
IRAT109	CK	1.50 ± 0.03 a	0.52 ± 0.04 a	0.29 ± 0.05 a	2.89 ± 0.16 b
	DS	1.33 ± 0.01 b	0.37 ± 0.04 b	0.30 ± 0.02 a	3.48 ± 0.16 a
HY73	CK	1.48 ± 0.03 a	0.56 ± 0.04 a	0.25 ± 0.01 a	2.66 ± 0.20 b
	DS	1.27 ± 0.01 b	0.37 ± 0.04 b	0.26 ± 0.01 a	3.37 ± 0.21 a
ANOVA	V	ns	ns	ns	**
	T	**	**	ns	**
	C × T	**	**	ns	**

Different letters indicate significant differences between the treatments using Tukey’s test (*p* < 0.05). Data represent means of n = 3 measurements ± standard deviation. “ns” means no significant difference, “**” means there is a significant difference at *p* < 0.01 level.

**Table 2:** Effects of drought stress on SOD, POD, and CAT activities and MDA content on day 9 after drought stress treatment.

Cultivar	Treatment	SOD (U/g)	POD (U/g)	CAT (U/g)	MDA (nmol/g)
HHZ	CK	167.9 ± 1.56 b	132.9 ± 1.39 b	75.2 ± 0.90 b	65.9 ± 0.31 b
	DS	218.7 ± 1.14 a	183.7 ± 1.02 a	102.5 ± 1.02 a	94.1 ± 1.26 a
IRAT109	CK	160.6 ± 1.88 b	125.6 ± 1.68 b	69.8 ± 0.71 b	57.0 ± 0.27 b
	DS	185.1 ± 3.02 a	150.1 ± 2.70 a	87.1 ± 1.37 a	75.8 ± 1.25 a
HY73	CK	111.5 ± 7.13 b	76.5 ± 2.38 b	68.1 ± 0.52 b	63.3 ± 0.72 b
	DS	138.2 ± 1.14 a	103.2 ± 1.02 a	91.2 ± 1.02 a	82.0 ± 0.42 a
ANOVA	V	**	**	**	**
	T	**	**	**	**
	C × T	**	**	**	**

SOD (superoxide dismutase); POD (peroxidase); CAT (catalase); and MDA (malondialdehyde). Different letters indicate significant differences between the treatments using Tukey’s test (*p* < 0.05). Data represent means of n = 3 measurements ± standard deviation. “**” means there is a significant difference at *p* < 0.01 level.

**Table 3:** Effects of drought stress on yield and yield components after drought stress treatment.

Cultivar	Treatment	Grain Yield (g/pot)	Panicle (no./pot)	Spikelets (no./panicles)	Seeding Rate (%)	1000-Grain Weight (g)
HHZ	CK	178.2 ± 5.43 a	61.7 ± 1.03 a	139 ± 1.48 a	81.1 ± 1.56 a	21.8 ± 0.24 a
	DS	124.4 ± 3.99 b	61.3 ± 1.37 a	116 ± 3.20 b	68.2 ± 1.58 b	20.6 ± 0.14 b
IRAT109	CK	172.5 ± 3.17 a	56.0 ± 0.89 a	116 ± 6.03 a	72.7 ± 0.91 a	31.5 ± 0.19 a
	DS	150.0 ± 3.51 b	55.0 ± 0.89 a	105 ± 2.64 b	69.4 ± 1.31 a	30.0 ± 0.18 b
HY73	CK	147.3 ± 3.01 a	41.0 ± 0.89 a	136 ± 1.49 a	72.4 ± 2.14 a	29.4 ± 0.29 a
	DS	120.5 ± 8.01 b	40.7 ± 1.37 a	120 ± 4.80 b	64.7 ± 1.30 b	29.3 ± 0.19 a
ANOVA	V	*	*	*	*	*
	T	**	ns	**	**	**
	C × T	**	ns	ns	**	**

Different letters indicate significant differences between the treatments using Tukey’s test (*p* < 0.05). Data represent the means of n = 3 measurements ± standard deviation. “ns” means no significant difference, “*” means there is a significant difference at *p* < 0.05 level, “**” means there is a significant difference at *p* < 0.01 level.

**Table 4:** Eigenvalue and cumulative contribution rate of each index for all three cultivars under drought stress at the booting stage.

Measured Index	Principal Component	
	PC1	PC2
Yield	0.281	0.371
Pn	0.307	0.251
Gs	0.305	0.241
LWP	0.324	−0.171
Chla/Chlb	−0.283	0.258
SPAD	0.289	0.419
MDA	−0.321	−0.112
Rubisco	0.296	−0.395
*Fv/Fm*	0.331	−0.055
*qP*	0.318	−0.033
*NPQ*	−0.252	0.545
Eigenvalue	8.493	1.165
Cumulative contribution rate (%)	77.21	87.8

## Data Availability

The original contributions presented in this study are included in the article; further inquiries can be directed to the corresponding authors.
